# Social inequalities in self-rated health in Sweden using the Population Health Performance Index: a cross-sectional study

**DOI:** 10.1093/eurpub/ckag017

**Published:** 2026-02-11

**Authors:** Rachel Sunny Inyangetuk, Miguel San Sebastián, Osvaldo Fonseca-Rodríguez

**Affiliations:** Department of Epidemiology and Global Health, Umeå University, Umeå, Sweden; Department of Epidemiology and Global Health, Umeå University, Umeå, Sweden; Department of Epidemiology and Global Health, Umeå University, Umeå, Sweden

## Abstract

Sweden faces challenges in improving average health and reducing health inequalities simultaneously. Traditional research methods often measure these aspects separately, posing a dilemma for policymakers. Thus, the Population Health Performance Index (PHPI) has been proposed as a summary measure that integrates both aspects. This study aimed to determine the PHPI for self-rated health (SRH) by income and education in Sweden across its 21 regions. Data were obtained from the 2021 Health on Equal Terms survey, a population-based cross-sectional study from Sweden. This survey included 17 538 participants aged 16–84 years. The outcome was poor SRH, stratified by the highest and lowest education and income groups. The PHPI was calculated as a weighted average of the mean and inequality index according to a neutral inequality aversion weight of 0.5. The prevalence of poor SRH was 28.7%, with pronounced disparities between high- and low-income/education groups. Overall, the PHPI by income and educational inequality was closer to zero across regions, albeit small relative differences were observed among the regions compared. The index also revealed substantial spatial disparities between Sweden’s northern and southern regions. The PHPI can be a complementary tool for policymakers to monitor social inequalities in health. The findings call for targeted interventions to address socioeconomic and regional disparities to achieve equity in health in Sweden.

## Introduction

Socioeconomic inequalities in health remain a persistent public health challenge in Sweden, challenging the egalitarian value of its welfare system, which aims to provide universal access to healthcare and social protection [1]. Although much effort has been devoted to monitoring and tackling health inequalities at the national and regional levels, there remain significant differences in health outcomes between people of high and low socioeconomic status [2]. An illustration of these disparities is the widening educational gap in health, particularly in obesity. Since the late 1960s, the prevalence of overweight and obesity has remained disproportionately higher among low-educated groups, with low-educated women at higher risk of obesity in early pregnancy [3]. Also, in a study of 20 European countries, Sweden had the largest difference in the relative risk of depression and diabetes between individuals with low and high educational levels [4]. Regarding economic differences, higher income levels have been associated with better 30-day survival after out-of-hospital cardiac arrest [5]; it is therefore not surprising that individuals with higher incomes increase their life expectancy at a faster rate than those with lower incomes [6]. Likewise, 50% of all psychiatric diagnoses, for both inpatient and outpatient care, have been found to be concentrated in the lowest income quintile of the population [7].

These inequalities persist despite notable improvements in overall population health. For instance, in Sweden, life expectancy has increased by an average of 9 years since the 1970s, while at age 30, a 3- to 6-year difference in life expectancy by educational levels has been observed [2]. Similarly, while cancer mortality in Sweden has decreased by 45%, a cohort study showed that all-site cancer mortality has increased among those with low levels of education [8]. The same trend is observed in self-rated health (SRH), as the proportion of people with good self-reported health has increased from 1980 to the present, but has declined among those with lower levels of education [9]. This paradox of improving average health while widening inequalities poses a significant challenge to population health goals that seek to improve overall health and eliminate inequalities [10].

Achieving these goals simultaneously is rare, as policymakers often trade off reducing inequalities against improving average health [11]. Additionally, there is a lack of consensus among policymakers and researchers about which measures best capture both means and inequalities, making it difficult to assess progress toward population health goals holistically [11, 12].

In response to this challenge, the Population Health Performance Index (PHPI) has been proposed by Kindig *et al*. as a summary measure to assess both overall health and inequalities within a population [11]. This index has previously been applied in a study examining under-five mortality disaggregated by wealth quintiles, using World Health Organization Equity Monitor data from Nepal, Ethiopia, Peru, Rwanda, and Zimbabwe (1994–2020) [13]. To the best of our knowledge, there is no published application of the PHPI in Europe, representing a significant gap in the literature. Therefore, this study is the first to apply the PHPI in a European context and aims to examine geographical variations in education and income inequalities in SRH, using the PHPI, in Sweden.

## Methods

This study utilized cross-sectional data, national representative data from the 2021 “Health on Equal Terms” (HET) survey in Sweden, with a target population aged 16-84 years. Calibration weighting was applied to account for both sampling probability and non-response, ensuring the representativeness of population-level estimates across socioeconomic groups and regions [14]. Using the personal identification number of the participants, the data were linked with national registers managed by Statistics Sweden to add their socioeconomic and demographic information [15].

This study had a population of 17 578 individuals. From this sample, people with missing information on education (*n* = 161), income (*n* = 60), or SRH (*n* = 154) were excluded. Thus, the final study population consisted of 17 203 individuals, representing 97.8% of the initial sample.

There are 21 decentralized regions in Sweden, with each responsible for providing health care services to its residents. Historically, the regions have been divided into three main geographical areas: Norrland (Northern Sweden), Svealand (Central Sweden), and Götaland (Southern Sweden). Norrland consists of the regions of Jämtland Härjedalen, Västerbotten, Västernorrland, and Norrbotten. It covers 58% of the country’s landmass but is home to only 12% of Sweden’s population. In contrast, Svealand, which is the smallest of the three areas, is home to Stockholm, which alone makes up 20% of the country’s population. Other regions in Svealand include Uppsala, Dalarna, Örebrö, Södermansland, Värmland, and Västmansland. Götaland, the most densely populated region, is home to 48% of Sweden’s population. It includes the regions of Skåne, Halland, Blekinge, Kronoberg, Kalmar, Jönköping, Västra Götaland, and Östergötland [16].

### Variables

The outcome, SRH, is a good and established predictor of mortality and morbidity, and a commonly used indicator of the population’s health. It is a widely used health measure for monitoring trends in health inequalities within and between the Nordic countries [17]. In this study, SRH was derived from participants’ response to the question “How do you assess your general state of health?” according to a five-point scale (very good; good; fair; poor; very poor). The response options were recoded and grouped into good (very good; good) and poor (fair; poor; very poor), in accordance with accepted standards for its classification [18, 19].

Two variables, education and income, were selected as indicators of socioeconomic position. Education was classified into three groups of low education (primary and lower secondary education), medium education (upper secondary education), and higher education (post-secondary education) according to the classification followed by Statistics Sweden [20]. Income, representing individual disposable income, is defined as the amount remaining for consumption or savings after paying taxes and accounting for all positive and negative transfers. Income was categorized into five quintiles, with quintile 1 representing the wealthiest group [21].

### Data analysis

Descriptive statistics were used to determine the prevalence of poor SRH in each region. Within each region, the prevalence of poor SRH was calculated separately for high and low education and income groups, and then the absolute differences by education and income were estimated.

The PHPI is a weighted average of two indices: the mean index and the inequality index.

First, the mean index for each region was calculated as follows:


$$\mathrm{Region}\;\mathrm{Mean}\;\mathrm{Inde}x_{i}=1-\left(\mathrm{Population}\;\mathrm{Mea}n_{i}/\mathrm{Population}\;\mathrm{Mea}n_{\mathrm{Most}\;\mathrm{Unhealthy}\;\mathrm{Region}}\right)$$

where the Population Mean is the prevalence of poor SRH in that particular region.

The mean index has a value that ranges between 0 and 1. A value of 1 represents the ideal scenario where no adverse health events occur, and 0 represents a group with the poorest outcome. In our context, a mean index of 1 would mean that no poor SRH was reported in a particular region, while a mean index of 0 would indicate the region/s with the highest prevalence of poor SRH. As suggested by the developers of the index, only the highest and lowest social groups should be considered; thus, the mean outcome is not a population mean but rather the mean of the lowest and highest education and income groups combined [11].

Second, we calculated the inequality index using the following formula for each region:


$$\mathrm{Region}\;\mathrm{Inequality}\;\mathrm{Inde}x_{i}=\;1-\left(\mathrm{Inequalit}y_{i}/\mathrm{Inequalit}y_{\mathrm{Most}\;\mathrm{Unequal}\;\mathrm{Region}}\right),$$

where Inequality represents the absolute social difference (the prevalence of poor SRH in high education/income groups minus the prevalence of poor SRH in low education/income groups).

Again, the inequality index ranges from 0 to 1, with a value of 1 representing no social inequality in the health outcome within a region and a value of 0 representing the most unequal region/s.

The PHPI is then calculated using a weighted average where the weights assigned to each component reflect the relative significance of mean and inequality measures. For instance, when equal importance is placed on the health mean and inequality within the chosen population, both indices are weighted equally at 0.5. Weights of w = 0.9, 0.75, 0.5, 0.25, and 0.1 correspond to strong, moderately strong, neutral, moderately weak, and weak aversion to the inequality, respectively [11]. For this study, equal weights of 0.5 were used for both the mean index and the inequality index. A sensitivity analysis was also done using weights of 0.25 and 0.75, and the results are presented in the Supplementary File.

The final PHPI formula can be represented as follows:


PHPI=(1-w)*Region Mean Index+w*Region Inequality Index.

A PHPI value of 1 represents the ideal scenario where a region is the healthiest (absence of poor SRH and social inequalities in SRH) relative to the worst region; however, it does not necessarily reflect whether the overall health status is good or bad. Conversely, the lowest possible value for the PHPI is 0, indicating that a region has both the least healthy mean population health (the highest prevalence of poor SRH) and the highest level of social inequality. The index therefore shows how regions or groups within a country perform relative to the worst-off and highlights differences between these groups, capturing both mean and inequalities at the same time [11].

The analysis was done using R version 4.2.3 [22].

### Ethical considerations

The study was approved by the Swedish Ethical Review Authority (approval no. 2021-02398).

## Results

The overall prevalence of poor SRH in Sweden was 28.7% and ranged from 22.2% to 34.8% across the regions, as shown in Table 1. The highest prevalence was observed in Norrbotten (34.8%) and Västernorrland (33.8%), and the lowest in Stockholm (22.2%) and Halland (23.8%). When looking at educational inequalities, the prevalence in the low-educated groups ranged from 34% in Jönköping to 44% in Blekinge (average prevalence of 39%). While among the highly educated groups, it ranged from 15.7% to 24.4% in Halland and Gotland, respectively, with a mean prevalence of 20%. The educational absolute difference ranged from 14 to 22 percentage points (pp), with a mean of 19 pp. In terms of income inequality, the prevalence in the lowest income quintile ranged from 27.5% to 37.5% in Halland and Jämtland, respectively (average prevalence of 33.3%). In the richest income quintile, the prevalence ranged from 15.6% in Stockholm to 22.2% in Norrbotten, with an average prevalence of 18.4%. The absolute difference by income ranged from 11 to 19 pp, with the average being 15 pp.

**Table 1.** Prevalence and absolute inequalities of poor self-rated health by education and income groups across regions

Region	*N*	Prevalence (%)	PLE (%)	PHE (%)	AIE (pp)	PLI (%)	PHI (%)	AII (pp)
Stockholm	3857	22	36	18	18	29	16	13
Uppsala	665	28	37	20	17	31	17	13
Södermanland	538	31	39	24	15	36	20	17
Östergötland	810	25	40	18	22	32	17	15
Jönköping	641	24	34	18	16	32	17	15
Kronoberg	361	24	35	19	17	31	18	13
Kalmar	418	28	37	23	14	33	16	17
Gotland	108	33	38	24	14	33	22	12
Blekinge	240	33	44	22	22	37	18	19
Skåne	2215	25	37	19	18	30	16	13
Halland	651	24	35	16	19	27	16	11
Västra Götaland	2877	27	38	19	19	32	17	15
Värmland	465	27	41	21	20	36	19	17
Örebro	454	31	41	21	20	34	20	13
Västmanland	444	31	41	22	19	34	18	16
Dalarna	509	27	41	20	21	35	19	15
Gävleborg	467	31	40	21	19	37	20	17
Västernorrland	426	34	39	20	19	36	19	17
Jämtland	203	33	43	24	19	38	20	18
Västerbotten	482	32	40	21	20	33	20	13
Norrbotten	402	35	43	21	22	35	22	13

PLE: prevalence in low education; PHE: prevalence in high education; AIE: absolute inequality in education; PLI: prevalence in low income; PHI: prevalence in high income; AII: absolute inequality in income.

**Figure 1.**
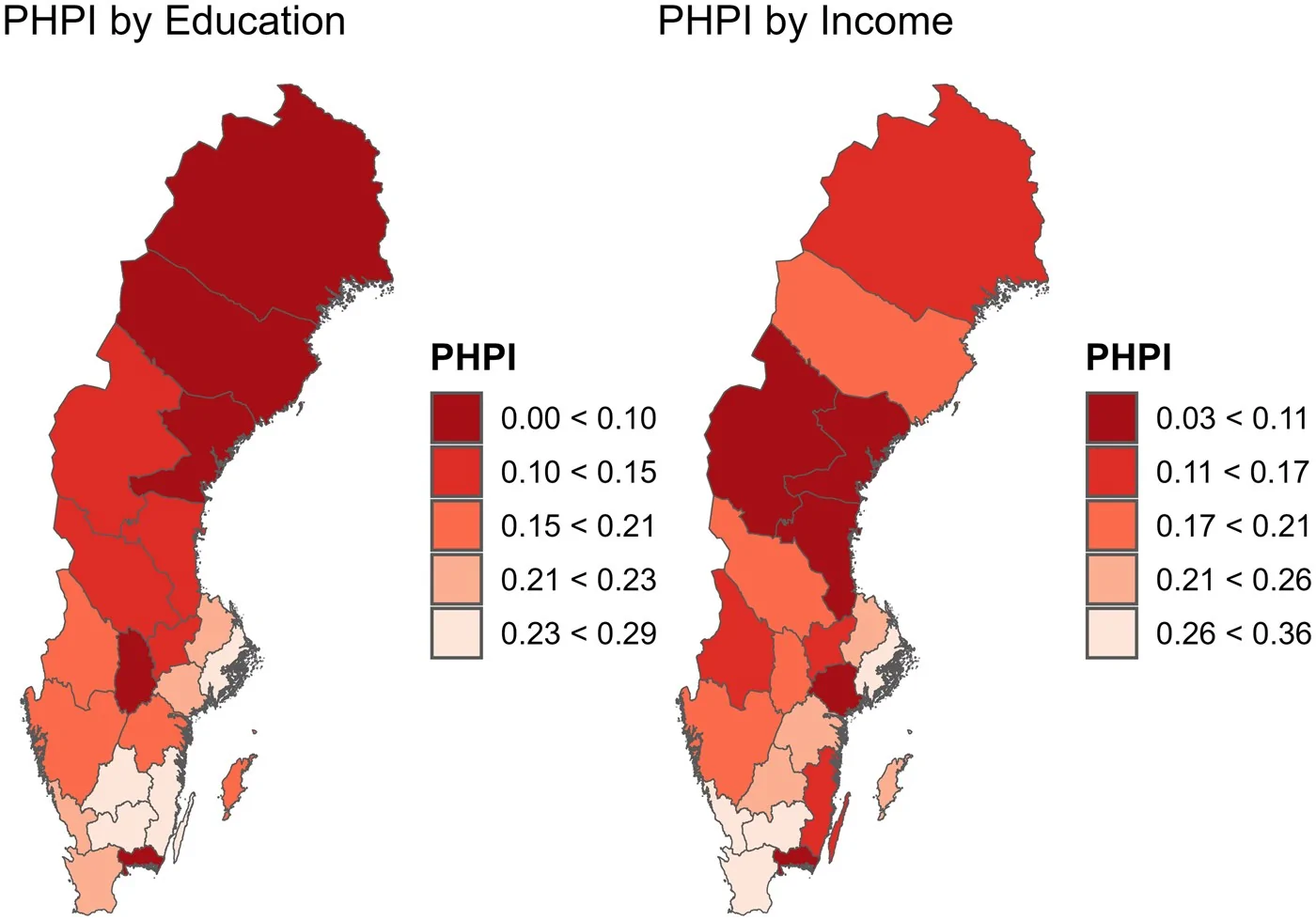
Spatial distribution of the PHPI by Education and Income Inequality across different regions in Sweden.

The results for the PHPI score are summarized in Table 2 and represented spatially in Fig. 1. The PHPI of poor SRH by education ranged from 0.00 in Norrbotten to 0.29 in Kalmar. In terms of income, the mean PHPI score ranged from 0.03 in Blekinge to 0.36 in Halland. The mean PHPI of poor SRH by education and income across the 21 regions was 0.17 and 0.19, respectively.

**Table 2.** PHPI by education and income inequality across different regions in Sweden

	PHPI of poor-SRH by education			PHPI of poor-SRH by income		
Region	Mean index	Inequality index	PHPI	Mean index	Inequality index	PHPI
Stockholm	0.36	0.19	0.28	0.36	0.29	0.33
Uppsala	0.2	0.23	0.21	0.2	0.29	0.24
Södermanland	0.1	0.34	0.22	0.1	0.1	0.1
Östergötland	0.29	0.02	0.16	0.29	0.22	0.26
Jönkoping	0.3	0.27	0.28	0.3	0.18	0.24
Kronoberg	0.3	0.26	0.28	0.3	0.29	0.3
Kalmar	0.21	0.36	0.29	0.21	0.1	0.16
Gotland	0.04	0.37	0.21	0.04	0.38	0.21
Blekinge	0.05	0	0.03	0.05	0	0.03
Skåne	0.27	0.17	0.22	0.27	0.28	0.28
Halland	0.32	0.14	0.23	0.32	0.4	0.36
Västra Götaland	0.21	0.14	0.17	0.21	0.21	0.21
Värmland	0.23	0.09	0.16	0.23	0.1	0.17
Örebro	0.1	0.1	0.1	0.1	0.28	0.19
Västmanland	0.12	0.15	0.14	0.12	0.16	0.14
Dalarna	0.24	0.06	0.15	0.24	0.17	0.21
Gävleborg	0.11	0.15	0.13	0.11	0.1	0.11
Västernorrland	0.03	0.15	0.09	0.03	0.08	0.05
Jämtland	0.07	0.16	0.11	0.07	0.05	0.06
Västerbotten	0.08	0.12	0.1	0.08	0.31	0.2
Norrbotten	0	0.01	0	0	0.32	0.16

The sensitivity analysis showed similar results to the main results (Supplementary Table S1), with Norrland having the lowest PHPI scores by education and income, regardless of inequality aversion levels, while the highest scores were observed in the southern regions.

## Discussion

This study represents the first application of the PHPI to assess the combined mean and inequality components of poor SRH in Sweden. The average PHPI score for poor SRH by income and education were 0.19 and 0.17, respectively. On average, Sweden performed slightly worse in the PHPI score by education than income, indicating that there are higher prevalences and inequalities in poor SRH by education. This finding aligns with previous research showing that in the Nordic countries, health inequalities are more strongly related to education than to income [23].

The prevalence of poor SRH in this study was 28.7%, consistent with previous findings [24–26] but slightly higher than those reported in other Nordic countries, such as Denmark (27%) and Norway (21.1%) [27]. Poor SRH was more common in lower educational and income groups, with almost twice the difference in prevalence compared to higher education and income groups. Similar finding has been reported in other European countries such as Denmark, Norway, and Austria [28, 29].

An enormous variation in the PHPI index, capturing both prevalence and social inequalities were observed across regions. Higher prevalence of poor SRH and greater inequalities were mainly found in northern Sweden, while southern regions such as Kronoberg and Halland achieved more than double the PHPI scores by education and income compared to the northern regions. Historically, regional health disparities between northern and southern Sweden have been well-documented, with northern Sweden experiencing higher prevalences in poor SRH, higher mortality rates, and lower life expectancy compared to the south [30]. These disparities may be driven by the growing income and educational inequalities between regions, leading to regional differences in health outcomes [31].

Lower levels of education are associated with reduced health literacy, fewer opportunities for higher-paying jobs, and health inequalities, all of which negatively impact SRH [32]. Education also serves as a determinant of an individual’s intellectual and material resources throughout their life course, shaping access to quality health services and influencing broader health outcomes [33, 34]. Nonetheless, disparities in educational attainment persist across regions. For example, in the southern region of Stockholm, approximately 22.6% of the population has attained post-secondary education of 3 years or more. In stark contrast, northern regions such as Norrbotten and Västernorrland exhibit considerably lower rates, with 13.6% and 13% of their populations, respectively, achieving similar educational levels [35].

The same regional disparities can also be seen in income levels, reflecting growing income inequalities, which have been linked to a higher prevalence of poor SRH [36]. Southern regions, like Stockholm and Halland, have significantly higher disposable incomes compared to northern regions such as Västernorrland and Västerbotten. These income disparities are further reflected in at-risk poverty rates, which are lower in Halland (9.8%) and Stockholm (10.4%) but notably higher in Västernorrland (13.5%) and Jämtland (13.8%) [37]. Income influences health both directly, by determining access to material resources, and indirectly, through behavioral and social factors [34].

Demographic differences may also play a role in the observed regional disparities in poor SRH. Southern regions in Sweden tend to have a younger population, whereas the north has a higher proportion of elderly residents [38]. This demographic disparity is important to consider because SRH levels typically decline with increasing age [39]. Additionally, the sparse population and long distances in Norrland create challenges in accessing healthcare, adversely affecting SRH. Poor or unsatisfactory healthcare accessibility has been found to be strongly associated with worse SRH, and residents who express a desire for improved healthcare access are more likely to report poorer health [40].

All the regions in this study had PHPI values closer to zero than to one, indicating that, even with equal weighting of the mean and inequality components, there is considerable room for improvement in the poor SRH by income and education.

### Strengths and limitations

The strength and originality of this study lie in the use of a nationally representative dataset and the use of a summary measure that captures both mean and inequality outcomes, thus effectively addressing population health goals. The PHPI can definitely be a good tool for monitoring social inequalities by geography or time.

Notwithstanding, it is important to note that although education and income were register-based, SRH was self-reported, which introduces the risk of recall bias, potentially leading to an over- or underestimation of the results.

A major limitation of the PHPI is that it does not differentiate whether poor health outcomes are concentrated among those with higher or lower socioeconomic status. Therefore, the PHPI should be used with caution and should not replace the examination of the mean and inequality components separately. These individual components may require distinct policy approaches, and ignoring them could hinder progress in improving average health or reducing inequalities [11]. Also, there is ambiguity regarding the optimal weight to apply, as varying weights based on users’ inequality aversion would yield different outcomes.

Focusing solely on relative performance compared to the worst-off group may not accurately reflect the overall health status of a population, as the PHPI only shows how regions perform relative to the worst-off region within the same country, which may still represent good health by international standards. In addition, the index does not allow comparisons beyond the specific context in which it is being used, as it should be interpreted in relation to the least advantaged group within the country or group being compared [11].

Finally, because the PHPI is constructed using weighted point estimates from the HET survey, it does not incorporate measures of statistical precision. As with any survey-based estimates, regional prevalence values are subject to sampling variability. Thus, differences between regions should be interpreted with caution.

## Conclusion

The PHPI is an innovative tool for policymakers to support informed decision-making to improve the average population health and reduce social inequalities in health. The PHPI in this study showed wide disparities in poor SRH, with northern regions experiencing both higher prevalence and greater health inequalities. These findings call for policymakers to address the existing regional differences in average and social inequalities in SRH by prioritizing improving access to healthcare, expanding educational opportunities, and addressing economic disparities, particularly in Norrland.

Future research could explore the possibility of using national averages for inequality and prevalence as reference points in calculating the PHPI, rather than highlighting disparities relative to the most disadvantaged region. This approach would measure how each region deviates from the national norm. It would also facilitate cross-country comparisons, as the index would not be anchored to a specific region’s performance but rather to an average that could be more globally contextualized. This approach could address the concern that not all regions can perform at the same level, even when overall health outcomes are excellent.

## Supplementary Material

ckag017_Supplementary_Data

## Data Availability

The data that support the findings of this study are not publicly available due to legal and ethical reasons. Key points The PHPI is an innovative tool that combines mean health and inequalities into a single metric, offering a more effective way to track progress in both improving overall health and reducing disparities. The PHPI scores in this study showed significant regional disparities, with Norrland having the lowest scores in the index by both income and education. Policymakers should prioritize reducing regional health inequalities by addressing socioeconomic disparities in education and income, particularly in Norrland.
