# Supplementary material for: Social inequalities in self-rated health in Sweden using the Population Health Performance Index: a cross-sectional study
Source: Eur J Public Health. 2026 Feb 11;36(2):ckag017. doi: 10.1093/eurpub/ckag017 (PMC13017663; doi:10.1093/eurpub/ckag017)
Supplement: ckag017_Supplementary_Data [file ckag017_supplementary_data.docx]

**Table S1**: **Sensitivity analysis**

|  | **PHPI of poor-SRH by Education** | | **PHPI of poor-SRH by Income** | |
| --- | --- | --- | --- | --- |
| **Region** | PHPI 0.25 | PHPI 0.75 | PHPI 0.25 | PHPI 0.75 |
| Stockholm | 0.32 | 0.23 | 0.34 | 0.31 |
| Uppsala | 0.21 | 0.22 | 0.22 | 0.27 |
| Södermanland | 0.16 | 0.28 | 0.10 | 0.10 |
| Östergötland | 0.22 | 0.09 | 0.27 | 0.24 |
| Jönkoping | 0.29 | 0.28 | 0.27 | 0.21 |
| Kronoberg | 0.29 | 0.27 | 0.30 | 0.29 |
| Kalmar | 0.25 | 0.32 | 0.18 | 0.13 |
| Gotland | 0.13 | 0.29 | 0.13 | 0.30 |
| Blekinge | 0.04 | 0.01 | 0.04 | 0.01 |
| Skåne | 0.25 | 0.20 | 0.28 | 0.28 |
| Halland | 0.27 | 0.18 | 0.34 | 0.38 |
| Västra Götaland | 0.19 | 0.15 | 0.21 | 0.21 |
| Värmland | 0.19 | 0.12 | 0.20 | 0.13 |
| Örebro | 0.10 | 0.10 | 0.15 | 0.24 |
| Västmanland | 0.13 | 0.14 | 0.13 | 0.15 |
| Dalarna | 0.19 | 0.10 | 0.22 | 0.19 |
| Gävleborg | 0.12 | 0.14 | 0.11 | 0.10 |
| Västernorrland | 0.06 | 0.12 | 0.04 | 0.06 |
| Jämtland | 0.09 | 0.13 | 0.06 | 0.06 |
| Västerbotten | 0.09 | 0.11 | 0.14 | 0.25 |
| Norrbotten | 0.00 | 0.01 | 0.08 | 0.24 |
